# A Metric-Based Few-Shot Learning Method for Fish Species Identification with Limited Samples

**DOI:** 10.3390/ani14050755

**Published:** 2024-02-28

**Authors:** Jiamin Lu, Song Zhang, Shili Zhao, Daoliang Li, Ran Zhao

**Affiliations:** 1 National Innovation Center for Digital Fishery, China Agricultural University, Beijing 100083, China; s20213081518@cau.edu.cn (J.L.);; 2 Key Laboratory of Smart Farming Technologies for Aquatic Animal and Livestock, Ministry of Agriculture and Rural Affair, Beijing 100083, China; 3 Beijing Engineering and Technology Research Centre for Internet of Things in Agriculture, China Agriculture University, Beijing 100083, China; 4 College of Information and Electrical Engineering, China Agricultural University, Beijing 100083, China

**Keywords:** fish recognition, few-shot classification, prototypical networks, attention mechanism

## Abstract

**Simple Summary:**

To address the challenge of limited sample size in fish data, we improved the prototypical networks to enhance model accuracy. In this study, an attention module was introduced on the basis of the prototypical networks, and improvements were made in the calculation of similarity. Across various fish datasets, our model achieved an accuracy improvement of 2% to 10% compared to the prototypical networks. This research has practical application value for scarce marine fish resources.

**Abstract:**

Fish species identification plays a vital role in marine fisheries resource exploration, yet datasets related to marine fish resources are scarce. In open-water environments, various fish species often exhibit similar appearances and sizes. To solve these issues, we propose a few-shot learning approach to identifying fish species. Our approach involves two key components. Firstly, the embedding module was designed to address the challenges posed by a large number of fish species with similar phenotypes by utilizing the distribution relationships of species in the embedding space. Secondly, a metric function was introduced, effectively enhancing the performance of fish species classification and successfully addressing the issue of limited sample quantity. The proposed model is trained end to end on fish species public datasets including the Croatian fish dataset, Fish4Knowledge and WildFish. Compared with the prototypical networks, our method performs more effectively and improves accuracy by 2% to 10%; it is able to identify fish effectively in small samples sizes and complex scene scenarios. This method provides a valuable technological tool for the development of fisheries resources and the preservation of fish biodiversity.

## 1. Introduction

Fish are an important global source of protein, so it is very important to explore and utilize fishery resources [1]. By identifying fish in the process of exploitation, we can learn about their distribution, and then understand the marine ecosystem and biodiversity. In recent years, significant progress has been made in the field of computer vision with the help of deep learning and large-scale modelling [2,3], which is of great academic and economic value for the study of fish identification methods. However, these methods require a substantial number of samples for each species for training, placing a significant constraint on the application of fish species identification. In contrast, inspired by the human capacity to identify new samples with just a few images, the concept of few-shot learning has been introduced, driving us to address the issue of fish species recognition.

Early methods of fish species identification relied on manual observation [4], which was time-consuming and laborious and affected research efficiency. With the development of computer vision and artificial intelligence [5,6], an efficient and automated underwater fish identification method has emerged [7]. Existing methods are mainly divided into two categories: machine learning [8,9,10] and deep learning [11,12,13]. Traditional machine learning methods require manual selection of features, such as support vector machines, clustering algorithms, and logistic regression. In contrast, deep learning methods can automatically learn more comprehensive and deeper features. However, deep learning methods are data-driven. Therefore, how we might learn effectively from limited samples has become the current focus of research.

In response to the above-mentioned issue, researchers have begun to investigate how to train image recognition models based on limited annotated samples. The recent emergence of few-shot learning methods aims to solve this problem and quickly establish cognitive ability to recognize new concepts with only a few examples. Relevant scholars are focused on completing few-shot learning tasks using the following three aspects:

(a) Augmenting data [14,15]: There are methods used to augment data manually. For example, on images, one can use panning, flipping, cropping, scaling and adjusting tone [15,16]. Due to the limited efficiency of the method of manually augmenting the data, autoaugmented search spaces [17] and a generative adversarial networks [18,19] have been designed to generate new samples. The translation model generates diverse and high-quality images for fish species with limited sample sizes [20].
(b) Optimizing the algorithm so that the target method can be quickly generalized to new tasks [21]: The two main approaches are optimization of existing parameters and meta-learning. In the strategy of optimizing existing parameters, the model is first trained using a large-scale dataset to obtain initial parameters and then fine-tuned in new tasks. Some classic algorithms include LSTD [22] and PATRON [23]. Meta-learning, on the other hand, improves the generalization performance of the base learner by iterating through the advanced meta-learner. Classic methods include MAML [24], meta-learner LSTM [25], and reptile [26]. The MAML-based reptile algorithm for fish recognition [27] outperforms classic deep learning methods.
(c) Metric learning, which a metric function is used to describe the similarity of two images: Firstly, the images are used to generate a feature space by embedding the model. For example, Siamese networks [28] share weights, prototypical networks [29] form category prototypes within the feature space, matching networks [30] build efficient learning networks using attention structures and memory-based networks, and cross-attention networks [31] introduce cross-attention modules. Secondly, in the feature space, categories can be distinguished based on the distance metric. For instance, prototypical networks [29] use learned category features for nearest-neighbor classification. CovaMNet [32] utilizes covariance representation for few-shot classification. In addition, methods such as the similarity metric [33] and image-to-class local metric [34] have also been proposed. The method proposed in this paper also falls within the realm of metric learning.

Deep learning models are complex and require large annotated datasets. Methods of few-shot fish recognition are limited in terms of data and accuracy, lacking generalizability. Underwater fish images face challenges such as complex backgrounds, poor image quality, and the similarity in fish morphology. To address these issues, we propose a small-sample fish recognition network that combines attention feature extraction with nearest-neighbor classification.

The main contributions of this paper are as follows:

(1) We have designed an attention prototype nearest neighbor network, which is a few-shot learning method distinct from traditional fish recognition approaches. This method can effectively solve the problem of limited sample size in fish identification.
(2) Our method can be used to generalize to new categories (categories not seen during training). Species classes can be identified by providing a small number of samples.
(3) The application of image recognition technology improves the efficiency of fisheries management, automatically identifies and monitors fish species, and achieves sustainable management of fisheries.

## 2. Materials and Methods

### 2.1. Materials

We selected three publicly available datasets for few-shot fish species recognition. The Croatian fish dataset [35] contains 794 images of 12 different fish species collected in the Croatia Adriatic. Fish4Knowledge [36] is a dataset of fish images obtained from long-term monitoring using underwater cameras and includes 23 fish species with 27,370 images. WildFish [37] is a large-scale benchmark for fish recognition in the wild, which includes 1000 fish species with a total of 54,459 images.

**Figure 1:**
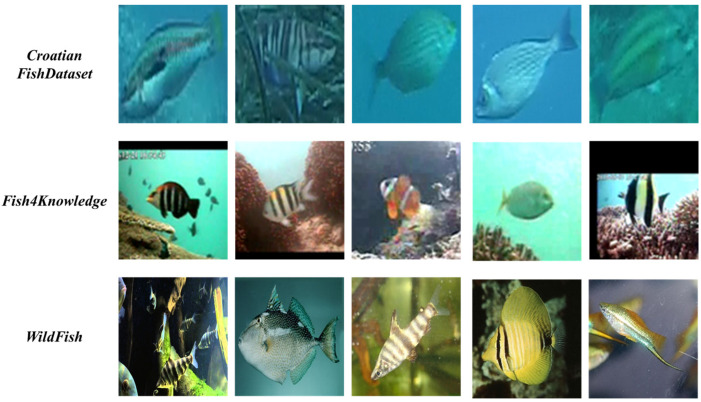
A random selection of images to demonstrate the image quality of the three datasets.

As shown in Table 1, the number of species, the number of images and the segmentation rate of the datasets are reflected for these three datasets. As shown in Figure 1, we randomly selected images from three datasets to demonstrate the image quality. We observed problems such as image clutter, complex scenes and unclear images, which pose a challenge for fish recognition. In our experiments, we set the resolution of the input image to 84 × 84 pixels. This paper adopts the K-way N-shot form to investigate the few-shot image classification task, which will be elaborated upon in Section 3.1, detailing the experiment setup.

### 2.2. The Proposed Method

**Figure 2:**
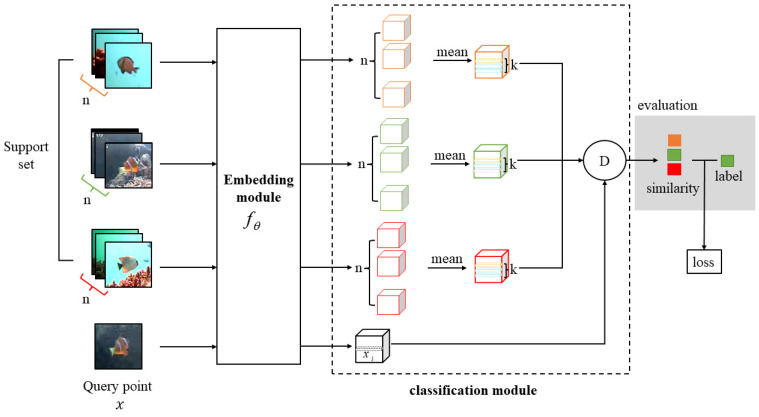
Overview of the framework of our method. n represents the number of images for each class in the support set. $x_{i}$ is the *i*th local descriptor of query point x. k denotes the k feature descriptors in the category prototype that are closest to the feature descriptor $x_{i}$ of the query point. The different colors represent different types of fish. In the diagram, orange represents Dascyllus reticulatus, green represents Amphiprion clarkia, and red represents Chaetodon trifascialis.

The framework of the method we propose is shown in Figure 2, consisting of two primary components: the embedding module and the classification module. The embedding module includes the base backbone and the attention mechanism module. The classification module incorporates prototypical networks and a nearest-neighbor classifier.

The specific implementation steps of this method are as follows:

Firstly, metric learning divides the dataset into support sets and query sets. The embedding module extracts image features from both the support set and query point, generating corresponding feature vectors. In the support set, each class has n images, and their averages are taken as class prototypes [29]. Secondly, by employing a nearest-neighbor classifier, the k closest feature descriptors, $x_{i}$, to the class prototypes in the query point, x, are identified. Then, the cosine distance is calculated to measure the similarity between the query set and the support set. The results of the similarity are compared with label information to obtain predictions. Finally, by continuously optimizing and updating the parameters during the training process, image classification is achieved.

#### 2.2.1. Embedding Module

**Figure 3:**
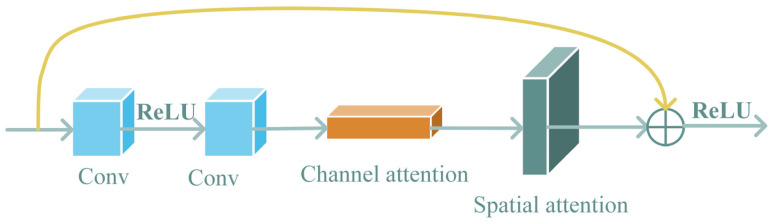
The overall structure of the hybrid attention mechanism.

In this section, we will describe the embedding module. To address the challenges of limited sample size, difficult feature extraction, and overfitting caused by network redundancy, we introduced an attention mechanism into the baseline network ResNet18 [5]. Feature extraction can map fish images into a vector space that can express rich semantics, thus improving the model’s accuracy. The hybrid attention mechanism, as shown in Figure 3, comprises both channel attention and spatial attention mechanisms. Firstly, the channel attention identifies important information in the fish images. Secondly, the spatial attention locates areas with the most information collection, enabling a more accurate localization of the primary region in the fish images.

**Figure 4:**
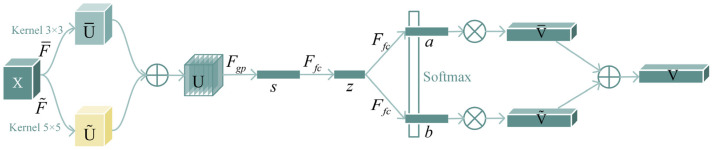
The structure of the channel attention mechanism. The channel attention tells the network what to pay attention to, focusing on useful information on the image.

(a) Channel attention mechanism:

The channel attention mechanism filters fish images to capture key information and improve model performance. The channel attention module is shown in Figure 4.

Firstly, 3 × 3 and 5 × 5 convolution kernels are selected to perform convolution operation on feature map X to generate $\bar{U}$ and $\tilde{U}$ respectively, that is, $\bar{F}:X\to \bar{U}\in R^{C\times H\times W}$ and $\tilde{F}:X\to \tilde{U}\in R^{C\times H\times W}$ (*H* and *W* denote the height and width of the image, and *C* denotes the channel count of the image.), where $\bar{F}$ and $\tilde{F}$ includes convolution, normalization and activation function. ReLU is used as the activation function. In order to improve the computational efficiency, the 5 × 5 convolution kernel is replaced by a 3 × 3 kernel and a dilated convolution with dilation size 2.

Secondly, the $\bar{U}$ and $\tilde{U}$ branches above are added element by element, that is, $U=\bar{U}\;+\;\tilde{U}$. Then, the global average pool is used to obtain the global information to obtain s, and the feature is compressed by the fully connected layer to obtain z. $s_{c}$ represents the average value of features in the c dimension. *d* refers to the feature dimension of the compressed model. In order to study the impact of *d* on the efficiency of the model, we use the reduction rate *r* to control, and *L* represents the minimum value of *d*, which is generally valued 32.

(1) $$s_{c}=F_{gp}(U_{c})=\frac{1}{H\times W}\sum_{i=1}^{H}\sum_{j=1}^{W}U_{c}(i,j)$$

(2) d=max(C/r,L)

Then, the feature dimension of z after squeeze is restored to *C* by two full connection layers, and the results after the two full connections are put together (this can be considered a matrix of *C* × 2); subsequently, a softmax operator is applied on the channel-wise digits (each column) of this matrix:

(3) $$a_{c}=\frac{e^{A_{c}z}}{e^{A_{c}z}+e^{B_{c}z}},\;b_{c}=\frac{e^{B_{c}z}}{e^{A_{c}z}+e^{B_{c}z}}$$

where *A*, *a* and *B*, *b*, respectively, represent the soft attention vectors of $\bar{U}$ and $\tilde{U}$. $A_{c}$ is row *c* of *A*, and $a_{c}$ is the *c*th element of a. $B_{c}$ and $b_{c}$ function in the same way.

Finally, the feature mapping *V* is obtained from the attention weights of the different convolution kernels, where $V_{c}$ denotes the feature mapping in the *c*th dimension.

(4) $$V=\left[V_{1},V_{2},\cdots ,V_{c}\right],V_{c}\in R^{H\times W}$$

(5) $$V_{c}\;=\;a_{c}{\bar{U}}_{c}+b_{c}{\tilde{U}}_{c},\;a_{c}+b_{c}=1$$

**Figure 5:**
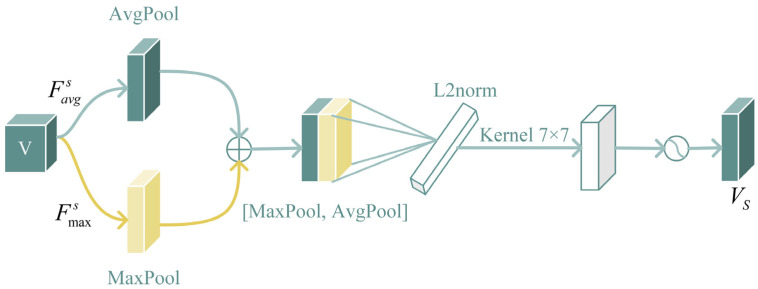
The structure of the spatial attention mechanism. The spatial attention tells the network where to pay attention, focusing on the key information in the image.

(b) Spatial attention mechanism:

Spatial attention mechanisms excel in discerning crucial information within feature space, resulting in robust classification performance, especially advantageous for handling fish images with limited samples. The spatial attention module is shown in Figure 5. Firstly, average pooling and max pooling of feature map *V* are performed to generate two maps $F_{avg}^{s}$ and $F_{\max}^{s}$, and then they are connected to generate efficient feature descriptors. Next, we compress the vectors using an L2-norm layer and normalize the contribution of each variable to ensure that the feature vector is not affected by the scale of each variable, thereby reducing the loss caused by the uneven distribution of fish samples. Finally, a 2D spatial attention map is generated through a 7 × 7 convolution kernel. It can be described as follows:

(6) $$\begin{array}{ll} M_{s}(F) & =\sigma (f^{7\times 7}\left\|\left(\left[AvgPool\left(F\right);MaxPool\left(F\right)\right]\right)\right\|)\\ & =\sigma (f^{7\times 7}\left\|\left(F_{avg}^{s},F_{\max}^{s}\right)\right\|)=\sigma (W\left\|\left(F_{avg}^{s},F_{\max}^{s}\right)\right\|+b) \end{array}$$

where σ represents the sigmoid function, ‖z‖ said 2-norm, $f^{7\times 7}$ said filter size of 7 × 7 convolution operation, and *W* and *b* the learning parameters.

Since the operation of fully connected layer and global average pooling layer will cause unrecoverable information loss, the feature extraction network does not contain the last fully connected layer and global average pooling layer. Suppose a fish image *X* is given, and an *h* × *w* × *d* tensor is generated by embedding the module (*h*, *w* and *d*, respectively, refer to the height, width, and dimension after convolutional feature mapping). It can be regarded a local descriptor of *m* (*m* = *h* × *w*) *d* dimensions, and its expression is as follows:

(7) $$f_{\phi }(X)=\{x_{1},x_{2},\cdots ,x_{m}\}$$

where $x_{i}$ is the *i*th local descriptor. In our experiment, given an image with a resolution of 84 × 84, we can obtain *h* = *w* = 6 and *d* = 512, which means that each image has a total of 36 local descriptors.

#### 2.2.2. Classification Module

In this section, we will describe the classification module, which consists of a prototype network and a nearest neighbor classifier.

Prototype network: The prototype network [29] is a simple and efficient few-shot learning method for image classification tasks involving learning the metric space. The images in the support set and query set are extracted by the embedding module and denoted $f_{\phi }(x)$. The learnable parameters of the shared network are ϕ, including the structure of the model, the initial parameters, and the learning rate, among others. There are n images for each class in the support set, and the average of them is taken as the class prototype, with $c_{k}$ denoting the prototype of k classes.

(8) $$c_{k}=\frac{1}{\left|S_{k}\right|}\sum_{i=1}^{n}f_{\phi }(x_{i})$$

$S_{k}=\left\{\left(x_{1},y_{k}),(x_{2},y_{k}),\cdots ,(x_{n},y_{k}\right)\right\}$ denotes a set of examples with class k in the support set. $y_{k}$ denotes the true label of class k.

Nearest-neighbor classifier: Feature extraction of the image through the embedding module will highlight the main area of the fish. At this time, the feature vector value of the main body of the fish is greater than the feature vector value of the background. Then, the distance of the query fish image from each fish category prototype is determined based on the k nearest feature descriptors among the feature descriptors of each fish category prototype corresponding to each feature descriptor in the feature information of the query fish image. The classification module we use is the nearest neighbor classifier, which calculates the distance between the query fish image and the fish category using k-NN.

Specifically, after a query set image *x* passes through the embedding module, $f_{q}(x)=\{x_{1},x_{2},\cdots ,x_{m}\}$ is generated. Meanwhile, after the support set image passes through the embedding module, the category prototype is obtained. In Equation (8), $c_{k}$ represents the category prototype. We then find the *k* values ${{\tilde{x}}_{i}^{j}|}_{j=1}^{k}$ in the category prototype that are nearest neighbors to each descriptor $x_{i}$ ( i∈[1,2,⋯,m]) of the query image. Finally, we calculate the distance between $x_{i}$ and ${\tilde{x}}_{i}$. The formula of cosine similarity is shown in Equation (10), which represents the distance between the query fish image and each fish category of the support set:

(9) $$d(f_{q}(x),c_{k})=\sum_{i=1}^{m}\sum_{j=1}^{k}\cos(x_{i},{\tilde{x}}_{i}^{j})$$

(10) $$\cos(x_{i},{\tilde{x}}_{i})=\frac{x_{i}^{T}{\tilde{x}}_{i}}{\left\|x_{i}\right\|\cdot \left\|{\tilde{x}}_{i}\right\|}$$

For a query image *x*, the nearest neighbor classifier produces a softmax distribution of labels over the support categories. The probability of predicting the query image *x* as the *t*-th class is as follows:

(11) $$p_{q}(y=t|x)=\frac{\exp(-d(f_{q}(x),c_{t}))}{\sum_{t’}\exp(-d(f_{q}(x),c_{t’}))}$$

Meanwhile, we train our model using cross-entropy loss to minimize the loss function of the real class m within the Adam optimizer.

(12) $$\begin{array}{ll} l & =-\log p_{q}(y=t|x)\\ & =d(f_{q}(x),c_{t})+\log\sum_{t’}\exp(-d(f_{q}(x),c_{t’})) \end{array}$$

## 3. Experiments and Results

### 3.1. Experiment Setup

Experimental configuration: The experiments were executed on a server with the following configuration: two CPUs (Intel(R) Xeon(R) Silver 4210R, Hewlett Packard, Chongqing, China), four RAMs (32 G), and one GPU (NVIDIA GeForce RTX 3090, NVIDIA, Santa Clara, CA, USA, with 24 GB). PyTorch was used as the deep learning framework for this experiment. In Table 2, we list the experiment parameters. An Adam optimizer was employed with an initial learning rate of 0.001 and a batch size of 128. The model underwent training for 100 epochs, each comprising 600 training episodes and 600 validation episodes. Testing was conducted for 20 epochs. Hyperparameters were validated and adjusted based on the validation set after the experiment’s conclusion.

Episode composition: Few-shot classification usually splits the dataset into a training set (including validation set) and test set, denoted Dtrain and Dtest (Dtrain ∩ Dtest = Ø). Unlike traditional deep learning dataset splitting, the training and test sets include the support set and the query set. A subset of K categories is randomly selected from the training set to form a category subset, denoted Dts. N images are randomly selected from each class of Dts as the support set and M images as the query set, with different image samples selected for the support and query sets. The selection process of the test set is similar. In general, we consider K-way N-shot classification, where k-way means that the support set has k classes and n-shot means that there are n samples per class.

In [25,30], a simple method is used to construct episodes. For example: five way, five shot, and one query refer to one episode. The specific process is as follows: five classes are randomly selected, and five images are randomly selected from these five classes as the support set. Then, one image that has no intersection with the support set is randomly selected as the query point. In the testing phase, 600 episodes were randomly selected and evaluated by top-one average accuracy. This process was repeated 20 times and the results were finally reported in terms of average accuracy. In addition, we provided 95% confidence intervals, which are indicated by the “±” symbol in the experimental results.

Evaluation protocols: The classification performance of the few-shot model was evaluated using the fish dataset. During testing, we treated one episode as a classification evaluation, where a class prototype vector was computed from the embedding vector in the support set, and then class prediction was performed in the query set by the nearest neighbor classifier. Our classification accuracy evaluation metrics are defined as follows:

(13) $$A\mathrm{ccuracy}=\frac{T}{T+F}$$

where *T* (True) is the sample that was correctly predicted, and *F* (False) is the sample that was incorrectly predicted.

### 3.2. Model Comparison

In this section, prototypical networks [29], relation networks [33], DN4 [32], CAN [31], RENet [38], and our method are compared. The experimental results are shown in Table 3; it can be seen that the accuracy of our method on one shot and five shots is higher than that of the prototypical network, relation network, DN4, CAN and RENet.

As the Croatian fish dataset has only three test categories, the model accuracy can only be tested on three categories for this dataset. For the Croatian fish dataset, our method improved the accuracy by 12.111% in a one-shot setting and 6.235% in five-shot setting as compared with the prototypical network. For the Fish4Knowledge dataset, compared with the original prototypical network, our method greatly improved from 62.286% to 78.873% in the one-shot experiment, and from 78.032% to 88.592% in the five-shot experiment, being slightly lower than DN4 and RENet. For the WildFish dataset, our method improves slightly over the prototypical network, DN4 and RENet, but significantly improves over the relational network and CAN.

### 3.3. Ablation Experiment

#### 3.3.1. Comparison of Different Shots

**Figure 6:**
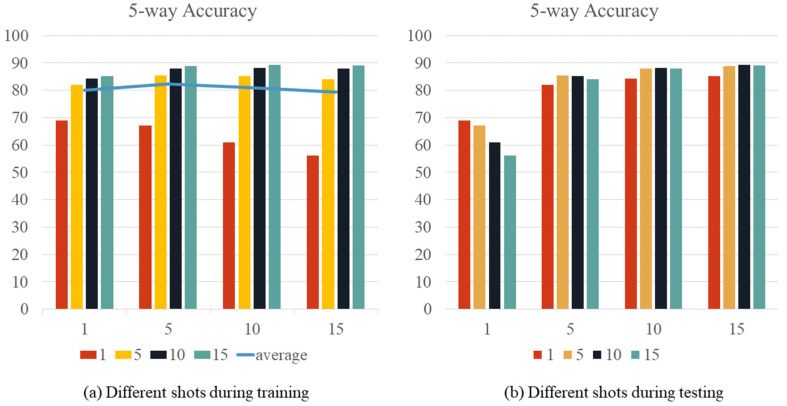
The accuracy results for different values of shot.

In this section, we experimentally evaluate the effect of different shots on fish classification performance. The Wildfish dataset was chosen for this experiment due to its diversity. Four different shots were selected and compared in this study. During the training phase, we changed the shot values and fixed the number of episodes. The models were then tested under the same test conditions. Then, five-way N-shot (N = 1, 5,10, 15) tasks were performed on the fish datasets using our proposed method. Accuracy results are shown in Figure 6. As shown in Figure 6a, the average accuracy is higher when N = 5 shots than when N = 1, 10, and 15. Therefore, the best performance is achieved at N = 5 shots. In Figure 6b, the best results are usually achieved when the training and test shots are equal.

#### 3.3.2. Performance Comparison of Embedding Module

Firstly, we investigated the effect of backbone network on fish species recognition. In Table 4, we compare four commonly used networks Conv-64F, VGG16, Resnet12, and Resnet18.

As can be seen in Table 4, the model using Resnet18 outperforms Conv-64F, VGG16, and Resnet12 in our experiments and has significant advantages. Resnet18 has a more complex structure compared to other networks. Therefore, we use Resnet18 as the base backbone network to extract features from images.

Next, we investigated the effect of the attention mechanism on the embedding module. In Table 5, we compare the performance of ResNet18 improved by the hybrid attention mechanism with the original ResNet18 in terms of classification accuracy. We found that adding either the channel or the spatial attention module improved accuracy, with the combination of the two providing the most significant improvement. In addition, our proposed hybrid attentional mechanism provides higher accuracy than CBAM to accurately locate the main regions of an image and to solve the problem of classifying subtle differences between different fish species.

#### 3.3.3. Performance Comparison of Distance Functions

In order to investigate the effect of distance function on image classification, we compare three commonly used distance functions: Manhattan, cosine and Euclidean. The classification accuracies of different distance functions are shown in Table 6. The model using Euclidean distance performs significantly better than the other models on the fish4knowledge dataset. We compared the Manhattan vs. cosine vs. Euclidean distance and 5-way vs. 15-way training episodes in the 1-shot and 5-shot scenarios. We note that the 15-way episode achieves higher accuracy than the 5-way episode.

#### 3.3.4. Performance Comparison of Different Modules

We verified the effectiveness of our proposed module through ablation experiments and the results are presented in Table 7. It is encouraging to note that our approach achieves better results compared to the prototype network. This is due to the use of the attention mechanism module, which contains valuable information that significantly enhances the performance of the CNN architecture. CNNs refer to convolutional neural networks, which are capable of efficiently learning and extracting hierarchical features for a more comprehensive understanding of image information. Our proposed nearest-neighbor classifier also demonstrates superior performance over the prototype network. Comprehensive experimental results show that the introduction of the attention mechanism module combined with the nearest neighbor classification module can significantly improve the classification accuracy.

### 3.4. Visualization Results for Significant Areas

**Figure 7:**
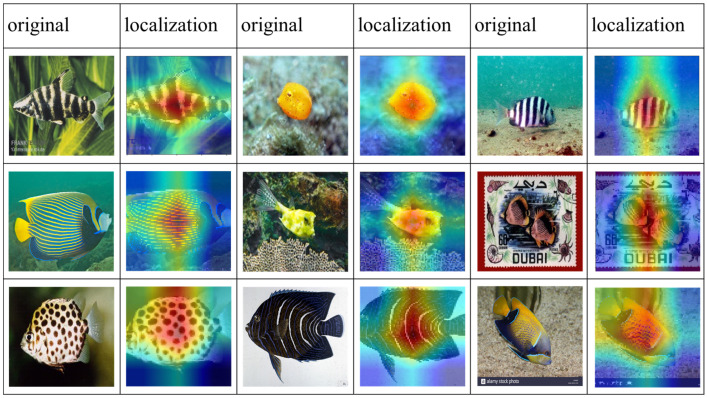
Class activation mapping visualization.

Class activation maps [41] were used to observe the region of the image where the embedding module was focused. In some cases, the model may focus more on the central part of the fish, while in others, it may focus more on the edges. This depends on the characteristics of the training data and the architecture of the model. In our work, we chose the model to focus on the central part of the fish because in our task, this part contains richer information. As shown in Figure 7, our proposed feature extraction network can locate the subject region in image recognition.

### 3.5. Generalization Performance Analysis

**Figure 8:**
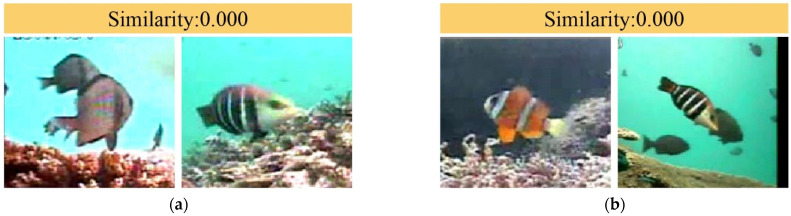

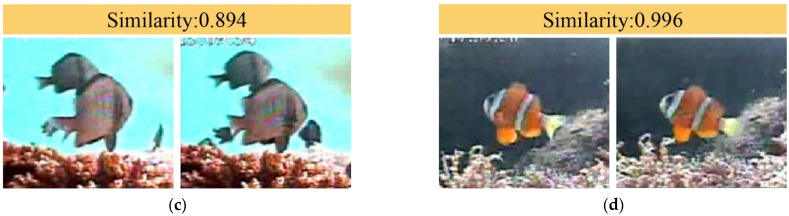
The similarity of the two input pictures obtained by our method. (**a**) the similarity of the two graphs is 0.000, (**b**) the similarity of the two graphs is 0.000, (**c**) the similarity of the two graphs is 0.894, (**d**) the similarity of the two graphs is 0.996.

The attention prototype nearest neighbor network we proposed demonstrates excellent generalization performance. Since the training and test sets are not intersected, we used data not involved in training for testing and determined the classification results by comparing the similarity of the input images. The test results are shown in Figure 8. We can find that the similarity of Figure 8a,b is 0, the similarity of Figure 8c is 0.894, and the similarity of Figure 8d is 0.996. The similarity of fish images of the same category is closer to 1, whereas that of different categories is closer to 0. The results show that our method can generalize to new categories, perform well in new categories, and has high generalization performance.

## 4. Conclusions

In this paper, a few-shot learning method for the problem of fish classification in complex underwater environments is proposed. The problem of difficult feature extraction of underwater targets is effectively solved by introducing the attention mechanism. The problem of uneven distribution of target objects due to odd sample data is solved by the normalization layer. By using local descriptors for nearest neighbor classification, the classification error caused by few samples is reduced, and finally the classification accuracy of underwater fish is improved. Our proposed attention prototype nearest neighbor network performed well on three publicly available datasets and showed a significant improvement in accuracy of ~10% compared to the prototype network. This approach is valuable in the field of aquaculture, especially for fish images with limited samples, providing an important reference for academic and practical applications.

Image recognition technology can provide insights into the distribution, abundance and behaviors of fish in ecosystems, leading to sustainable management of fisheries. In the future, we will develop a computer application that will help fisheries scientists to use our model to easily identify fish species.

## Figures and Tables

**Table 1:** Basic information about datasets. Different datasets are divided into different proportions. There is no intersection of train, val and test. The validation set is mainly used to verify whether the model is overfitted and adjust the training parameters, and the test set is used to evaluate the model results after training. Therefore, the ratio of val and test is set to a smaller value.

Fish Data	Categories	Images (Total)	Dataset Split (Train–Val–Test)
Croatian fish dataset [35]	12	794	7:2:3
Fish4Knowledge [36]	23	1038	16:2:5
WildFish [37]	1000	54,459	640:160:200

**Table 2:** The summary of ad hoc parameters and learnt parameters.

Hyperparameter Name	Set Value
lr	0.001
gamma	0.5
weight_decay	0.0005
batch_size	128
image_size	84
train_epoch	100
test_epoch	20
train_episode	600
test_episode	600

**Table 3:** Comparison of three-way classification on the Croatian fish dataset and five-way classification on the Fish4Knowledge and WildFish datasets with metric learning algorithms. Bold text indicates the best result in a given column.

Method	3-Way Accuracy (%)		5-Way Accuracy (%)			
	Croatian Fish Dataset		Fish4Knowledge		WildFish	
	1-Shot	5-Shot	1-Shot	5-Shot	1-Shot	5-Shot
ProtoNet [29]	63.268 ± 0.308	78.135 ± 0.219	62.286 ± 0.211	78.032 ± 0.182	65.947 ± 0.275	83.456 ± 0.199
CAN [31]	65.584 ± 0.252	72.161 ± 0.225	70.058 ± 0.213	80.586 ± 0.156	55.676 ± 0.291	69.013 ± 0.255
DN4 [32]	67.137 ± 0.239	80.410 ± 0.219	71.697 ± 0.223	89.467 ± 0.131	66.540 ± 0.276	84.476 ± 0.194
RelationNet [33]	55.083 ± 0.296	77.644 ± 0.222	51.647 ± 0.213	70.587 ± 0.202	40.575 ± 0.246	74.818 ± 0.231
RENet [38]	69.283 ± 0.527	80.600 ± 0.204	74.818 ± 0.231	**89.617 ± 0.200**	64.667 + 0.106	77.678 ± 0.253
ours	**75.379 ± 0.272**	**84.370 ± 0.176**	**73.873 ± 0.173**	88.592 ± 0.112	**68.944 ± 0.241**	**85.372 ± 0.165**

**Table 4:** Results of proposed model with different settings of backbone network. Bold text indicates the best result in a given column.

Method	3-Way Accuracy (%)		5-Way Accuracy (%)			
	Croatian Fish Dataset		Fish4Knowledge		WildFish	
	1-Shot	5-Shot	1-Shot	5-Shot	1-Shot	5-Shot
Conv-64F	61.797 ± 0.224	76.377 ± 0.207	60.810 ± 0.273	73.742 ± 0.236	61.208 ± 0.228	72.742 ± 0.236
VGG16 [39]	**68.766 ± 0.152**	76.401 ± 0.161	**63.140 ± 0.204**	76.661 ± 0.181	62.686 ± 0.179	82.936 ± 0.181
Resnet12 [5]	53.317 ± 0.178	64.160 ± 0.183	58.766 ± 0.152	66.401 ± 0.161	62.686 ± 0.179	72.936 ± 0.181
Resnet18 [5]	63.268 ± 0.308	**78.135 ± 0.219**	62.286 ± 0.211	**78.032 ± 0.182**	**65.947 ± 0.275**	**83.456 ± 0.199**

**Table 5:** Comparison of classification accuracies between ResNet18 and the backbone network with the addition of the attention mechanism on the Croatian Fish dataset and Fish4Knowledge and WildFish datasets. CBAM is the convolutional block attention module proposed in [40]; ca is the channel attention module; sa is the spatial attention module; and ha is the hybrid attention mechanism. Bold text indicates the best result in a given column.

Method	3-Way Accuracy (%)		5-Way Accuracy (%)			
	Croatian Fish Dataset		Fish4Knowledge		WildFish	
	1-Shot	5-Shot	1-Shot	5-Shot	1-Shot	5-Shot
Resnet18	63.268 ± 0.308	78.135 ± 0.219	62.286 ± 0.211	78.032 ± 0.182	65.947 ± 0.275	83.456 ± 0.199
Resnet18 + CBAM	69.078 ± 0.296	**79.545 ± 0.198**	67.195 ± 0.201	83.400 ± 0.156	65.413 ± 0.277	82.235 ± 0.206
Resnet18 + ca	64.063 ± 0.236	78.199 ± 0.228	66.392 ± 0.222	80.239 ± 0.226	63.146 ± 0.243	82.358 ± 0.229
Resnet18 + sa	65.415 ± 0.277	78.831 ± 0.209	65.457 ± 0.275	83.155 ± 0.204	66.810 ± 0.273	82.742 ± 0.236
Resnet18 + ha	**69.181 ± 0.258**	79.197 ± 0.149	**67.698 ± 0.197**	**83.718 ± 0.142**	**67.624 ± 0.274**	**83.524 ± 0.200**

**Table 6:** Results of the proposed model with different settings of distance functions. Bold text indicates the best result in a given column.

Distance	5-Way Accuracy (%)		15-Way Accuracy (%)	
	1-Shot	5-Shot	1-Shot	5-Shot
Manhattan	59.335 ± 0.245	74.685 ± 0.193	73.146 ± 0.243	82.358 ± 0.229
Cosine	60.857 ± 0.203	73.933 ± 0.171	70.734 ± 0.215	81.894 ± 0.156
Euclidean	**71.332 ± 0.208**	**83.718 ± 0.142**	**73.912 ± 0.245**	**87.946 ± 0.141**

**Table 7:** Results from different modules. ha is the hybrid attention mechanism, and nn is the nearest-neighbor classifier. Bold text indicates the best result in a given column.

Method	3-Way Accuracy (%)		5-Way Accuracy (%)			
	Croatian Fish Dataset		Fish4Knowledge		WildFish	
	1-Shot	5-Shot	1-Shot	5-Shot	1-Shot	5-Shot
ProtoNet [29]	63.268 ± 0.308	78.135 ± 0.219	62.286 ± 0.211	78.032 ± 0.182	65.947 ± 0.275	83.456 ± 0.199
our-ha	69.181 ± 0.258	79.197 ± 0.149	67.698 ± 0.197	83.718 ± 0.142	67.624 ± 0.274	83.524 ± 0.200
our-nn	70.379 ± 0.272	79.685 ± 0.193	71.332 ± 0.208	84.370 ± 0.176	68.326 ± 0.179	84.400 ± 0.156
**our**	**75.379 ± 0.272**	**84.370 ± 0.176**	**73.873 ± 0.173**	**88.592 ± 0.112**	**68.944 ± 0.241**	**85.372 ± 0.165**

## Data Availability

The original contributions presented in the study are included in the article.
